# Thymic Involution and Function in People With HIV and General Population Controls: A Cross‐Sectional Analysis From Two Danish Cohort Studies

**DOI:** 10.1111/acel.70723

**Published:** 2026-09-23

**Authors:** Manon C. Vanbellinghen, Anders Boyd, Andreas Ohrt Johansen, Moises Alberto Suarez‐Zdunek, Andreas Dehlbæk Knudsen, Thomas Skårup Kristensen, Jørgen Tobias Kühl, Per Ejlstrup Sigvardsen, Michael Huy Cuong Pham, Andreas Fuchs Larsen, Klaus Fuglsang Kofoed, Børge Grønne Nordestgaard, Shoaib Afzal, Neeltje Kootstra, Susanne Dam Nielsen, Peter Reiss

**Affiliations:** ^1^ Department of Infectious Diseases Amsterdam UMC, Location University of Amsterdam Amsterdam the Netherlands; ^2^ Amsterdam Institute for Immunology and Infectious Diseases Amsterdam the Netherlands; ^3^ Department of Global Health Amsterdam UMC, Location University of Amsterdam Amsterdam the Netherlands; ^4^ Amsterdam Institute for Global Health and Development Amsterdam the Netherlands; ^5^ Department of Infectious Diseases, Inselspital Bern University Hospital, University of Bern Bern Switzerland; ^6^ Department of Cardiology Copenhagen University Hospital ‐ Rigshospitalet Copenhagen Denmark; ^7^ Viroimmunology Research Unit, Department of Infectious Diseases Copenhagen University Hospital ‐ Rigshospitalet Copenhagen Denmark; ^8^ Department of Radiology Copenhagen University Hospital‐Rigshospitalet Copenhagen Denmark; ^9^ Department of Cardiology Copenhagen University Hospital ‐ Amager & Hvidovre Hospital Hvidovre Denmark; ^10^ Department of Clinical Medicine, Faculty of Health and Medical Sciences University of Copenhagen Denmark; ^11^ Department of Clinical Biochemistry Copenhagen University Hospital ‐ Herlev and Gentofte Herlev Denmark; ^12^ Department of Experimental Immunology Amsterdam UMC, Location University of Amsterdam the Netherlands; ^13^ Department of Infectious Diseases Copenhagen University Hospital ‐ Rigshospitalet Copenhagen Denmark

**Keywords:** aging, HIV, immunosenescence, T cell, thymus gland

## Abstract

Thymic involution, the age‐related replacement of thymic soft tissue by fat, has been linked to immunosenescence and chronic inflammation, processes implicated in aging‐associated comorbidities. Untreated HIV infection is associated with thymic atrophy and reduced thymic output. Although thymic function may normalize after initiation of antiretroviral therapy, the long‐term consequences of HIV infection on thymic involution in people with HIV (PWH) remain unknown. We investigated whether thymic involution differs between PWH and population controls, and how its association with markers of T‐cell differentiation and inflammation in PWH. We conducted a cross‐sectional analysis including 654 PWH aged ≥ 40 years from the Copenhagen Comorbidity in HIV Infection study and 637 age‐ and sex‐matched population controls from the Copenhagen General Population Study. Thymic tissue was graded on a 4‐point scale based on adipose‐to‐soft tissue proportion on chest CT scans, with higher grades reflecting more soft tissue (i.e., less involution). Thymic output was assessed by signal joint T‐cell receptor excision circle (sjTREC) content in buffy coats. PWH had higher odds of a higher thymus grade compared to population controls (adjusted‐OR = 1.75, 95% CI = 1.31–2.33), after adjustment for age, sex, pack‐years, BMI, waist‐to‐hip ratio, physical activity, and alcohol intake. Higher thymus grade was associated with greater sjTREC content, and among PWH with higher proportions of naïve CD4^+^ and CD8^+^ T cells, lower proportions of senescent T cells, and lower IL‐6 and hs‐CRP levels. Our findings indicate thymic involution may be attenuated in some PWH on long‐term treatment, and that the preserved thymic output may help mitigate chronic inflammation.

## Graphical abstract

Thymic involution, the age‐related replacement of thymic soft tissue by fat, has been linked to immunosenescence and chronic inflammation. Whether HIV infection affects thymic involution remains unclear. We found that people with HIV on long‐term antiretroviral therapy had more thymic tissue than controls, indicating less thymic involution. More thymic tissue was associated with higher thymic output (sjTREC), and, among people with HIV with higher naïve CD4+ and CD8+ T‐cell proportions, fewer senescent T cells, and lower IL‐6 and hs‐CRP levels.

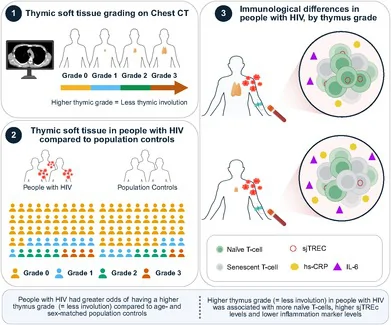

## 1 Introduction

The thymus is the central lymphoid organ where lymphoid progenitor cells undergo T‐cell receptor rearrangement and differentiate into mature naïve T cells. After puberty, thymic lymphoid tissue is gradually replaced by adipose tissue, a process known as thymic involution. The rate of involution varies between individuals and generally progresses faster in men, smokers, and individuals with a higher body mass index (BMI) (Araki et al. 2016; Sandstedt et al. 2023). Thymic involution contributes to T‐cell aging and chronic inflammation, processes implicated in the development of aging‐related comorbidities (Elyahu and Monsonego 2021; Liu et al. 2023; Thomas et al. 2020).

In people with HIV (PWH), studies have shown that the amount of thymic lymphoid tissue correlates with higher levels of naïve CD4^+^ T‐cells, higher markers of thymic output, and a more diverse T‐cell receptor repertoire (Kolte et al. 2009; Rb‐Silva et al. 2019; Rubio et al. 2002). Over time, preserved thymic output may help mitigate immune activation and chronic inflammation associated with HIV infection, potentially reducing the risk of aging‐related comorbidities. Indeed, one study in older PWH on long‐term antiretroviral therapy (ART) found that more thymic soft tissue on chest computed tomography (CT) scans, considered to represent lymphoid tissue, was associated with a lower prevalence of metabolic syndrome and frailty (Guaraldi et al. 2019). Additionally, in a similar population of older PWH, those with a biomarker profile reflecting higher thymic output had fewer comorbidities over time (Vanbellinghen et al. 2024).

Studies investigating the effects of HIV infection on the thymus have shown that untreated HIV infection disrupts the thymic microenvironment and is associated with thymic atrophy and reduced thymic output (Autran et al. 1996; Bonyhadi et al. 1993; Douek et al. 2001; Stanley et al. 1993). Some studies suggested that thymic function may be normalized after initiation of ART, although no studies have examined thymic involution in long‐term treated HIV infection compared with general population controls, and the consequences of HIV infection on thymic involution in this population therefore remain unknown (Dion et al. 2004; Douek et al. 1998). Despite the lack of evidence, the prevailing assumption is that thymic involution is accelerated in people with HIV relative to the general population, leading to premature immunosenescence (Deeks 2011; Gaardbo et al. 2012; Montano et al. 2022; Velardi et al. 2021). However, it is possible that HIV infection may result in compensatory attenuation of thymic involution compared to its normal age‐related trajectory. We therefore hypothesized that having HIV infection would be associated with less thymic involution than in general population controls of similar age.

We aimed to investigate whether adult PWH on long‐term ART have a higher prevalence of thymic soft tissue than general population controls, and to identify HIV‐related factors associated with the presence of thymic soft tissue among PWH. To do so, we conducted a cross‐sectional analysis in two well‐characterized cohorts of PWH and age‐ and sex‐matched general population controls, combining CT‐based thymus grading with peripheral markers of thymic output, specifically T‐cell receptor excision circle (TREC) content. In PWH, we further examined the association between thymic grade and markers of T‐cell differentiation, immune activation, and systemic inflammation.

## 2 Methods

### 2.1 Study Design and Participants

The Copenhagen Comorbidity in HIV Infection (COCOMO) study is a prospective observational cohort investigating non‐AIDS comorbidities in people with HIV (ClinicalTrials.gov: NCT02382822). People with HIV were recruited from the HIV outpatient clinics of Copenhagen University Hospital—Rigshospitalet or Amager‐Hvidovre between March 2015 and November 2016. Detailed study procedures have been previously described (Ronit et al. 2016). Controls without HIV were selected from the Copenhagen General Population Study (CGPS), a prospective cohort study of the general Danish population initiated in 2003, designed to investigate prevalent population‐based morbidities. In 2010, CT imaging was added to the research protocol of the CGPS for participants aged 40 years and above.

For this analysis, we included people with HIV and population controls if they were 40 years or older, had an available research, non‐contrast chest CT scan, and a cryopreserved buffy coat sample. PWH were frequency matched by sex (male, female) and 5‐year age strata (10 strata from 40 to 89 years) to participants from the CGPS. CT scans were reviewed and individuals with missing scans, scans of insufficient quality, signs of median sternotomy, suspected thymoma, or other pathological changes of the thymus were excluded. We intended a 1:1 matching ratio and did not replace participants after post‐matching exclusions. Of the 1099 PWH enrolled in the COCOMO study, 666 met inclusion criteria and were matched to 666 population controls from the CGPS. Following revision of the CT scans, 12 PWH and 29 population controls were excluded, resulting in a final study population of 654 PWH and 637 population controls. Details of participant selection are provided in Figure S1.

Ethics approval was obtained by the Regional Ethical Committee of Copenhagen (COCOMO: H‐8‐2014‐004; CGPS: H‐KF‐01‐144/01). The study was performed in accordance with the Declaration of Helsinki.

### 2.2 Clinical Assessments

Identical questionnaires were used in both cohorts to collect information regarding medical history, smoking, alcohol consumption, and medication use. Trained clinical staff conducted a comprehensive physical examination in both cohorts, which included anthropometric and blood pressure assessments. HIV‐related variables, including current and nadir CD4^+^ T‐cell counts, were retrieved from patient records (Ronit et al. 2016).

Hypertension was defined as blood pressure ≥ 140/90 mmHg and/or current use of antihypertensive treatment; diabetes as non‐fasting plasma glucose ≥ 11.1 mmol/L, or glycated hemoglobin ≥ 48 mmol/L or use of antidiabetic agents; dyslipidaemia as low‐density lipoprotein ≥ 3 mmol/L or use of lipid‐lowering drugs; central obesity as waist circumference ≥ 80 cm in women and ≥ 94 cm in men. eGFR was calculated using the 2021 Chronic Kidney Disease Epidemiology Collaboration (CKD‐EPI) equation.

### 2.3 Radiological Assessments

CT imaging for both cohorts was performed using a 320‐multidetector scanner (Aquilion One Vision Edition, Canon Medical Systems, Japan) at Copenhagen University Hospital—Rigshospitalet. Tube voltage (100–120 kV) and current (280 and 500 mA) were chosen based on BMI.

Non‐contrast chest CT scan images were visually assessed in the axial plane using external workstations (Vitrea 7.14; Vital Images Inc., Minnetonka, MN, USA). Two readers (M.V. and A.O.) graded all CT scans, blinded to the HIV status of participants. Thymic tissue was visually graded on a 4‐point scale (0 to 3) based on the proportion of adipose and soft tissue, with higher grades reflecting less thymic involution (Ackman et al. 2013). Grades were defined as follows: complete fatty replacement with no identifiable soft tissue in the thymic bed (Grade 0), predominantly fatty replacement of the thymus (Grade 1), approximately one half (between 40% and 60%) fatty tissue and one‐half (between 40% and 60%) soft tissue (Grade 2), and predominantly soft tissue (Grade 3). Cases with suspected thymoma or other pathological changes to the thymus were reviewed in consultation with a board‐certified radiologist (T.K.) and excluded, if confirmed. We assessed inter‐rater agreement by randomly selecting 100 CT scans and having each of the reviewers (M.V. and A.O.) rate them independently. Agreement was determined using weighted Cohen's Kappa. We assessed intra‐rater agreement by randomly selecting 25 CT scans per reviewer and having each of the reviewers (M.V. and A.O.) evaluate their respective set three times, separated by at least a one calendar day interval. Agreement was determined using the intraclass correlation coefficient (ICC), derived from a linear mixed‐effects model with thymus grade as the outcome and participant number as random effect. None of the scans selected for inter‐ and intra‐rater agreement were used more than once. In participants with a thymus Grade of 1 or higher, thymic attenuation and size were measured on the axial image with the maximum anterior–posterior (AP) diameter of the thymus. Attenuation was measured in Hounsfield Units (HU) by manually tracing a region of interest (ROI) along the thymic bed, excluding as much of the surrounding mediastinal fat as possible, and the mean attenuation within the ROI was recorded. Thymic size was assessed by measuring the AP diameter and transverse diameter.

### 2.4 Laboratory Assessments

Lipids, creatinine, glucose, glycated hemoglobin (HbA1c), and high‐sensitivity C‐reactive protein (hs‐CRP) were measured in non‐fasting venous blood samples. A standardized protocol was used for buffy coat extraction in PWH and population controls. In short, EDTA‐anticoagulated venous blood was kept on ice until centrifugation at 4°C to obtain plasma and buffy coats. Buffy coats were transferred to 1 mL MAT‐3741 2D screw tubes (Fisher Scientific, Hampton, NH, U.S.), with DMSO added as a cryoprotective agent, and subsequently stored at −80°C. All blood samples were processed and analyzed at the same laboratory located at Copenhagen University Hospital—Herlev and Gentofte.

### 2.5 Signal Joint T‐Cell Receptor Excision Circle (sjTREC) Content Determination

Buffy coats were shipped on dry ice to the Department of Experimental Immunology (University of Amsterdam, The Netherlands). Total DNA was isolated from buffy coats (stored at −80°C) using the QIAamp DNA blood mini kit (Qiagen, Venlo, the Netherlands) according to the manufacturer's instructions. sjTREC content was determined by nested PCR analysis as previously described (Ferrando‐Martínez et al. 2010). In the primary PCR, primer pair DTF6 AGAAGGCTCTGTCTAGTGTG and DTR61 TCTGACATTTGCTCCGTG were used to amplify sjTREC content in 5 ng of DNA, using Platinum Taq‐polymerase (Invitrogen) under the following PCR conditions: pre‐incubation: 95°C for 2 min (4.8°C/s); amplification: 25 cycles of 95°C for 20 s (4.8°C/s), 57°C for 45 s (2.5°C/s), and 72°C for 30 s (4.8°C/s); final extension at 72°C for 5 min (4.8°C/s); hold at 4°C (2.0°C/s). In the secondary PCR, sjTREC content was then quantified using 6 μL of the primary reaction as input in a secondary PCR with Platinum‐Taq polymerase (Invitrogen), primer pair DTF7 AGGCTCTGTCTAGTGTGATAAC and DTR66 TGACATGGAGGGCTGAAC, and SYBR Green (Invitrogen) for detection. To ensure amplification specificity, a melting curve analysis was performed for every sample with a positive SYBR Green signal and compared with that of a positive control. The following qPCR program was used: pre‐incubation: 95°C for 10 min (4.8°C/s); amplification: 45 cycles of 95°C for 10 s (4.8°C/s), 57°C for 20 s (2.5°C/s), and 72°C for 15 s (4.8°C/s); melting‐curve: 95°C for 5 s (4.8°C/s), 65°C for 1 min (2.5°C/s), melt from 65°C to 97°C (0.06°C/s); Stage 4, cooling at 40°C for 10 s (2.5°C/s). A plasmid containing the first‐round PCR product was used as a standard curve to calculate the sjTREC content (Figure S2). PCR analyses were performed on the LightCycler 480 system (Roche). sjTREC copy numbers were calculated using the LightCycler 480 software. The lower limit of detection (LLD) was defined as the lowest sjTREC level consistently detectable in the patient samples. Based on the performance of the assay in our sample set, the LLDwas set at 5 log sjTREC copies/ng DNA. TRECs are episomal DNA circles formed during T‐cell receptor gene recombination in the thymus and are not duplicated during mitosis, and their peripheral abundance is therefore considered a proxy of thymic output (Middelkamp and Kekäläinen 2025).

### 2.6 Laboratory Assessments in People With HIV

Plasma concentrations of the innate immune activation markers soluble CD14 (sCD14) and soluble CD163 (sCD163) were measured using enzyme‐linked immunosorbent assays (ELISA; R&D Systems). Plasma concentration of the cytokine interleukin‐6 (IL‐6) was measured using a multiplex electrochemiluminescence assay (Meso Scale Discovery, MSD). Cytomegalovirus (CMV) IgG concentrations were measured in plasma using the Diasorin LIASON CMV IgG II. CMV IgG seropositivity was defined as concentrations ≥ 14 U/mL, according to the manufacturer's instructions.

### 2.7 Collection of PBMC and Flow Cytometry Analysis in People With HIV

Technical procedures for PBMC collection and flow cytometry analysis in the COCOMO cohort have been described previously (Hove‐Skovsgaard et al. 2020; Ronit et al. 2016). Briefly, peripheral blood mononuclear cells (PBMCs) were isolated from heparinized blood at cohort enrolment using density gradient centrifugation within one hour of venipuncture and cryopreserved in a medium containing 10% DMSO. Preparation of the cell suspension and aliquoting into cryovials were performed at 4°C. Cells were frozen using a stepwise temperature decrease to −80° using CoolCell cell freezing containers (BioCision, CA, United States) and then transferred to liquid nitrogen within 72 h. For the flow cytometric analysis, cryopreserved PBMCs were thawed in 37°C water, resuspended and incubated overnight at 37°C and 5% CO_2_. The PBMCs were then washed, resuspended, and stained for flow cytometry. The following antibodies were used for cell surface staining: CD3 PerCP, CD4 BV510, CD8 FITC, CD28 PE‐Cy7, CD57 APC, CD38 PE‐Cy7, HLA‐DR APC, CCR7 Pacific Blue, CD45RA PE, CD25 PE‐Cy7, and CD127 FITC. Fixable Viability Stain 780 (FVS780) (BD) was used to determine viability. T‐cell subsets were defined as naive (CD45RA^+^ CCR7^+^), regulatory (CD25^+^CD127^low), senescent (CD28^−^CD57^+^), and activated (HLA‐DR^+^CD38^+^) within CD4^+^ or CD8^+^ T‐cells. Acquisition was performed on a BD FACSCanto II and gated by the same operator using FlowJo v. 10.3 (BD). The gating strategy has been described previously (Hove‐Skovsgaard et al. 2020). Viability was a median of 75% (interquartile range [IQR] 69–87).

### 2.8 Statistical Analysis

We assessed differences between groups using the Student's *t*‐test, Kruskal‐Wallis, one‐way ANOVA, or Pearson's χ^2^ test, as appropriate.

Correlations between thymus grade (≥ 1) and thymic attenuation or size (AP and transverse diameters) were assessed using the Kendall's tau‐b (*τ*_*b*_) correlation coefficient. To assess whether thymus grade corresponded with thymic output, the correlation between sjTREC content (log sjTREC copies/ng DNA) and thymus grade was evaluated using Kendall's *τ*_*b*_. Additionally, we performed a Tobit regression of log‐transformed sjTREC content on thymus grade as a continuous variable to, with left‐censoring at the LLD, estimate the change in sjTREC content per level increase in thymus grade. We additionally extended this model to include HIV status and an interaction term between thymus grade and HIV status; differences in the slope by HIV status were assessed using a Wald χ^2^ test of the interaction term. For all sjTREC analyses, values below the lower limit of detection were set to the detection limit.

To assess the association between HIV status and thymus grade, we modeled the probability of belonging to each thymus grade using ordinal logistic regression. We included HIV as a covariate from which the univariable odds ratio (OR) and 95% confidence interval (CI), comparing the odds of overall increases in thymus grade between PWH and population controls, were calculated. In a multivariable model, we included the following covariates selected a priori based on biological plausibility and evidence from the literature as potential confounding variables: sex, age, BMI, pack‐years of smoking, ethnicity (European descent), waist‐to‐hip ratio, physical activity (very/moderately active vs. very/moderately inactive) and weekly alcohol intake (Araki et al. 2016; Jahan‐Mahin et al. 2025; Noppert et al. 2023; Rengifo et al. 2024; Sandstedt et al. 2023). We obtained adjusted‐OR and their 95% CI from this model. We removed any covariates with unstable parameter estimates (i.e., fewer than 15 observations in any category of the outcome), or that were collinear with other covariates (i.e., variance inflation factor above 5). Covariates which violated the proportionality assumption (i.e., *p* < 0.05 from the Brant test), were included using a partial proportional odds model, allowing these covariates to have non‐proportional effects across thymus grades. We tested goodness‐of‐fit using the modified Hosmer–Lemeshow test (Fagerland and Hosmer 2016).

To assess the associations between HIV‐related factors and thymus grade, we modeled the probability of belonging to each thymus grade using an ordinal logistic regression, including only PWH. We added the following HIV‐related factors individually in univariable analyses: time since HIV diagnosis, nadir CD4^+^ T‐cell count, prior AIDS‐defining illness, viral load at ART initiation, age at time of HIV diagnosis, CMV IgG concentration, current use of nucleoside reverse transcriptase inhibitors (NRTIs), non‐nucleoside reverse transcriptase inhibitors (NNRTIs), protease inhibitors (PIs), integrase strand transfer inhibitors (INSTIs), current or prior exposure to thymidine analogues (TA, i.e., stavudine and zidovudine) and/or to didanosine (ddI), and current or prior exposure to indinavir (IDV), lopinavir (LPV) and saquinavir (SQV). HIV‐related factors were individually adjusted for the demographic and lifestyle factors from the main multivariable model. Because most HIV‐related variables may reflect age‐ or cohort effects, we present models both with and without adjustment for age. Models for TA/ddI and IDV/LPV/SQV exposure were not adjusted for waist‐to‐hip ratio, as it was considered a potential mediator. In a *post hoc* analysis, we examined whether IDV/LPV/SQV exposure partly explained the association between TA/ddI exposure and thymus grade by including both in a multivariable model not adjusted for age.

In PWH with complete biomarker data, the distribution of T‐cell subsets, soluble markers (IL‐6, hs‐CRP, sCD14, and sCD163), and sjTREC content were summarized as medians (interquartile range, IQR) across thymus grades and were compared using the Kruskal‐Wallis test.

We conducted statistical analyses using R (version 4.2.1, Vienna, Austria). For all statistical tests, we set the level of significance at an *α* of 5%.

## 3 Results

Characteristics of PWH and population controls at the time of CT acquisition are presented in Table 1. CT scans were obtained between February 2015 and November 2016 in PWH and between June 2010 and December 2018 in population controls. The median age was 52 years (IQR = 47–61) for PWH and 53 years (IQR = 47–61) for population controls. Both groups consisted predominantly of men of European descent. Compared with PWH, population controls had a higher BMI, consumed more alcohol per week, and had a higher prevalence of dyslipidaemia. PWH were more often current smokers and had a higher waist‐to‐hip ratio. Among PWH (Table 2), the median age at HIV diagnosis was 37 years (IQR = 31–45), with a median time since diagnosis of 16 years (IQR = 8–23).

**TABLE 1:** Characteristics at time of CT acquisition of people with HIV and population controls.

	PWH (*n* = 654)	Population controls (*n* = 637)	*p*
Age, years	52 (47–61)	53 (47–61)	0.34
Male sex	558 (85%)	545 (86%)	0.90
European descent	559 (87%)	616 (98%)	< 0.0001
BMI, kg/m^2^	24.6 (22.4–27.3)	26.3 (24.2–28.6)	< 0.0001
Waist‐to‐hip ratio	0.94 (0.88–0.99)	0.92 (0.87–0.97)	< 0.0001
Waist circumference, cm	94 (87–103)	94 (87–102)	0.23
Hip circumference, cm	101 (97–106)	103 (98–108)	0.0003
Central obesity	378 (60%)	363 (58%)	0.58
Smoking status			< 0.0001
Never	217 (34%)	306 (49%)	
Former	253 (39%)	250 (40%)	
Current	173 (27%)	73 (12%)	
Pack‐years in ever smokers	20 (8–35)	15 (8–27)	0.0023
Weekly alcohol intake, grams	84 (24–162)	96 (48–168)	0.0057
Physical activity			0.027
Inactive	57 (9%)	38 (6%)	
Moderately inactive	222 (36%)	197 (31%)	
Moderately active	270 (43%)	318 (51%)	
Very active	76 (12%)	75 (12%)	
Diabetes	35 (5%)	25 (4%)	0.21
Hypertension	301 (48%)	284 (45%)	0.53
Dyslipidaemia	367 (55%)	433 (66%)	< 0.0001
eGFR, mL/min/1.73m^2^	92 (81–102)	95 (87–103)	< 0.0001
hs‐CRP, mg/L	1.20 (0.60–2.43)	1.01 (0.57–1.79)	0.0014

*Note:* Data are *n* (%), or median (IQR); continuous variables are presented as median (IQR), regardless of their distribution. Missingness in variables used in the regression models is provided in Table S1.

Abbreviations: BMI, body mass index; eGFR, estimated glomerular filtration rate; hs‐CRP, high‐sensitivity C‐reactive protein; IQR, interquartile range; PWH, people with HIV.

**TABLE 2:** HIV‐related variables at time of CT acquisition, overall and by thymus grade.

	All PWH (*n* = 654)	Thymus Grade 0 (*n* = 413)	Thymus Grade 1 (*n* = 123)	Thymus Grade 2 (*n* = 65)	Thymus Grade 3 (*n* = 53)	*p* ^a^
Age at HIV diagnosis, y	37 (31–45)	38 (32–47)	36 (30–43)	38 (29–41)	32 (26–38)	< 0.0001
Time since HIV diagnosis, y	16 (8–23)	17 (10–25)	14 (6–21)	10 (7–17)	14 (8–22)	< 0.0001
On cART	644 (99%)	404 (98%)	122 (99%)	65 (100%)	53 (100%)	0.81
HIV RNA < 200 copies/mL	640 (98%)	405 (98%)	120 (98%)	64 (98%)	51 (96%)	0.82
HIV RNA at start ART, log(copies/mL)	4.92 (1.05–1.36)	5.0 (4.4–5.6)	4.9 (4.4–5.3)	4.7 (4.1–5.2)	4.7 (4.2–5.2)	0.074
Prior AIDS‐defining illness	129 (20%)	87 (21%)	23 (19%)	11 (17%)	8 (15%)	0.65
CD4 nadir, cells/ μL	220 (100–323)	200 (83–310)	230 (100–370)	276 (218–440)	270 (181–345)	0.0002
CMV IgG seropositive	617 (95%)	390 (95%)	118 (96%)	59 (91%)	50 (94%)	0.49
CMV IgG concentration, U/mL	158 (121–493)	168 (129–525)	144 (113–371)	142 (115–292)	144 (116–177)	0.0003
PI use at time of CT	199 (31%)	128 (32%)	35 (29%)	18 (28%)	18 (34%)	0.85
NRTI use at time of CT	615 (95%)	383 (94%)	118 (97%)	61 (95%)	53 (100%)	0.28
NNRTI use at time of CT	310 (48%)	203 (50%)	57 (47%)	30 (47%)	20 (38%)	0.39
INSTI use at time of CT	183 (28%)	112 (28%)	36 (30%)	19 (30%)	16 (30%)	0.95
TA/ddI exposure^b^	379 (58%)	267 (65%)	66 (54%)	25 (38%)	21 (40%)	0.0001
IDV/LPV/SQV exposure^c^	240 (37%)	177 (42%)	36 (29%)	14 (22%)	13 (24%)	0.0002

*Note:* Data are *n* (%), or median (IQR); continuous variables are presented as median (IQR), regardless of their distribution. Missingness in variables used in the regression models is provided in the Table S2.

Abbreviations: AIDS, acquired immunodeficiency syndrome; cART, combination antiretroviral therapy; CMV, cytomegalovirus; ddI, didanosine; INSTI, integrase strand transfer inhibitor; IQR, interquartile range; NNRTI, non‐nucleoside reverse transcriptase inhibitor; NRTI, nucleoside reverse transcriptase inhibitor; PI, protease inhibitor; RNA, ribonucleic acid; U, units.

^a^ *p*‐values reflect comparisons across thymus grade groups.

^b^ Two participants were on zidovudine at the time of CT scan, all other exposures indicate prior use.

^c^ Six participants were on lopinavir, and two on saquinavir at the time of CT scan, all other exposures indicate prior use.

**FIGURE 1:**
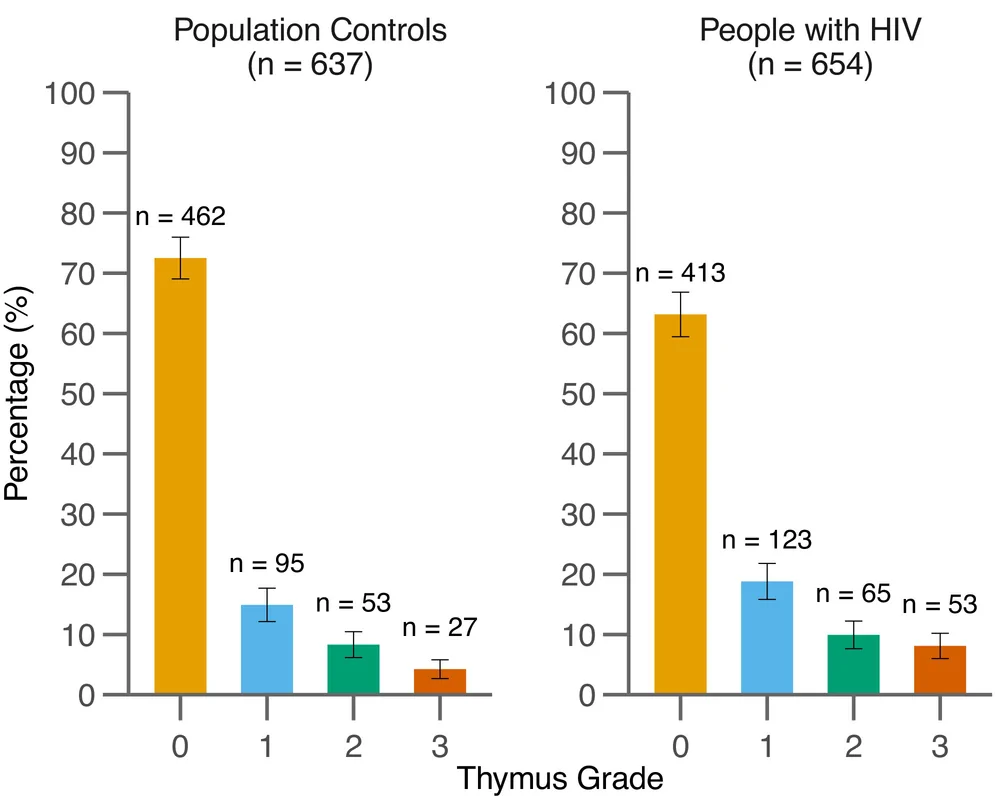
Distribution of thymus grade stratified by HIV status. Counts (*n*) above each bar indicate the number of individuals per thymus grade. Error bars represent the 95% confidence intervals.

The distribution of thymus grades differed significantly between PWH and population controls (*p* = 0.0013) (Figure 1). Among population controls, the majority had thymus Grade 0 (*n* = 462, 73%), while Grades 1, 2, and 3 were observed in 95 (15%), 53 (8%), and 27 (4%) participants, respectively. In PWH, thymus Grade 0 was observed in 413 (63%), with Grades 1, 2, and 3 present in 123 (19%), 65 (10%), and 53 (8%) participants, respectively. PWH had significantly higher odds of having a higher thymus grade (OR = 1.55, 95% CI = 1.23–1.96, *p* = 0.0002). This association remained significant after adjusting for age, sex, pack‐years of smoking, BMI, waist‐to‐hip ratio, physical activity, and weekly alcohol intake (adjusted‐OR = 1.75, 95% CI = 1.31–2.33, *p* = 0.0001) (Table 3). Missingness in variables used in the regression models is provided in Table S1.

**TABLE 3:** Association between HIV status and thymus grade, ordinal regression analysis.

	OR^a^ (95% CI)	*p*	aOR^b^ (95% CI)	*p*
HIV‐positive status	1.55 (1.23–1.96)	0.0002	1.75 (1.31–2.33)	0.0001
Age (per year)	0.89 (0.88–0.91)	< 0.0001	0.91 (0.89–0.93)	< 0.0001
Female sex	3.38 (2.51–4.56)	< 0.0001	1.61 (1.08–2.39)	0.018
European descent	0.39 (0.26–0.58)	< 0.0001	—	—
BMI (per kg/m^2^)	0.85 (0.82–0.88)	< 0.0001	0.89 (0.85–0.93)	< 0.0001
Waist‐to‐hip ratio (per 0.01)	0.88 (0.86–0.89)	< 0.0001	0.90 (0.88–0.92)	< 0.0001
Pack years (per 5 years)	0.90 (0.86–0.93)	< 0.0001	0.96 (0.92–1.00)	0.010
Weekly alcohol intake (per 10 g)	0.98 (0.97–0.99)	0.0001	1.00 (0.92–1.01)	0.10
Moderate/very physically active	1.60 (1.26–2.04)	0.0001	1.32 (1.00–1.74)	0.051

*Note:* Each row shows the association between that variable and thymus grade, from unadjusted (OR) and adjusted (aOR) ordinal regression models. In the adjusted model, 120 observations (97 PWH, 23 population controls) are omitted due to missingness of covariates. European descent was not included in the adjusted model, as only 12 participants of non‐European descent had thymus Grade 2.

Abbreviations: aOR, adjusted odds ratio; BMI, body mass index; OR, odds ratio; PWH, people with HIV.

^a^ Unadjusted.

^b^ Adjusted for age, sex, pack‐years, BMI, waist‐to‐hip ratio, physical activity, and weekly alcohol intake.

Inter‐rater agreement of thymus grading was high (weighted Cohen's κ = 0.88, 95% CI = 0.81–0.94), as was intra‐rater agreement (ICC = 0.90, 95% CI = 0.85–0.94). Representative CT‐images of each thymus grade are presented in Figure S3. Thymic attenuation and size measurements are presented in Table S2 A strong positive correlation was found between thymus grade and thymic attenuation (HU) (*τ*_*b*_ = 0.646, *p* < 0.0001), which was similar in PWH (*τ*_*b*_ = 0.648, *p* < 0.0001) and population controls (*τ*_*b*_ = 0.648, *p* < 0.0001) (Figure S4).

Buffy coats for sjTREC measurements were available in 627 (98%) of population controls and all 654 (100%) PWH. The overall median time between sampling and CT scanning was 59 days (IQR: 27–93), with a shorter interval in PWH (29 days; IQR: 14–58) than in population controls (86 days; IQR: 61–127). The proportion of participants with sjTREC content above the lower limit of detection was 20% (172/868) for thymus Grade 0, 40% (85/215) for Grade 1, 36% (42/118) for Grade 2, and 49% (39/80) in Grade 3. Among PWH, the proportion of participants with sjTREC content above the lower limit of detection was 20% for thymus Grade 0, 33% for Grade 1, 37% for Grade 2, and 51% for Grade 3. Among population controls, the corresponding proportions were 20%, 49%, 34%, and 44%, respectively. There was a significant, positive association between thymus grade and sjTREC content (*τ*_*b*_ = 0.21, *p* < 0.0001), indicating that participants with higher thymus grades had higher thymic output. From the Tobit regression, each one‐unit increase in thymus grade was associated with a 0.40 (95% CI = 0.30–0.51) increase in log sjTREC copies/ng DNA (*p* < 0.0001). The increase in log sjTREC copies/ng DNA per one‐unit increase in thymus grade did not differ by HIV status (*p* for interaction = 0.72): 0.45 (95% CI 0.30–0.61; *p* < 0.001) among PWH and 0.34 (95% CI 0.20–0.48; *p* < 0.001) among population controls.

HIV‐related variables are summarized across thymus grade in Table 2. Median age at HIV diagnosis and time since HIV diagnosis were highest in those with Grade 0 and tended to decrease as thymus grade increased. CD4 nadir was lowest in those with Grade 0 and higher in those with Grades 1, 2, and 3. While CMV serostatus did not differ between groups, CMV IgG concentration was significantly lower in those with higher thymus grades. The proportion of PWH with current or prior exposure to thymidine analogues was highest among those with thymus Grade 0 (65%) and decreased to 40% in those with Grade 3. Exposure to IDV/LPV/SQV was similarly highest in thymus Grade 0 and lower in those with Grades 1, 2, and 3.

Several HIV‐related variables were associated with thymus grade in the adjusted models (Table 4). Before additional adjustment for age, a higher CD4 nadir (adjusted‐OR per 50 cells/μL = 1.08, 95% CI = 1.02–1.14, *p* = 0.0005) was associated with higher thymus grade, while older age at HIV diagnosis (adjusted‐OR per 5 years = 0.84, 95% CI = 0.76–0.92, *p* = 0.0004) and longer time since HIV diagnosis (adjusted‐OR per 5 years = 0.88, 95% CI = 0.79–0.99, *p* = 0.031) were associated with lower thymus grade. TA/ddI (adjusted‐OR = 0.39, 95% CI 0.27–0.56, *p* < 0.0001) and IDV/LPV/SQV exposure (adjusted‐OR = 0.41, 95% CI 0.28–0.60, *p* < 0.0001) were also associated with lower thymus grade and remained so after additional adjustment for age. In the *post hoc* analysis including both exposures simultaneously (without adjustment for age), these associations were attenuated (adjusted‐OR = 0.52, 95% CI = 0.34–0.80, *p* = 0.0029, and adjusted‐OR = 0.58, 95% CI = 0.37–0.93, *p* = 0.022, respectively).

**TABLE 4:** Association between HIV related variables and thymus grade, ordinal logistic regression analysis PWH.

	OR (95% CI)	*p*	aOR^a^ (95% CI)	p‐value	aOR^b^ (95% CI)	*p*
Age at HIV diagnosis (per 5 years)	0.82 (0.75–0.88)	< 0.0001	0.84 (0.76–0.92)	0.0004	1.02 (0.91–1.16)	0.65
Time since HIV diagnosis (per 5 years)	0.82 (0.75–0.90)	< 0.0001	0.88 (0.79–0.99)	0.031	0.97 (0.86–1.10)	0.65
HIV RNA at start ART (per 1 log(copies/mL))	0.87 (0.73–1.03)	0.10	0.91 (0.76–1.11)	0.36	0.92 (0.75–1.12)	0.41
Prior AIDS‐defining illness	0.78 (0.52–1.15)	0.22	0.68 (0.41–1.10)	0.13	0.80 (0.50–1.31)	0.38
CD4 nadir (per 50 cells/μL)^c^	1.09 (1.04–1.13)	0.0001	1.08 (1.02–1.14)	0.005	1.04 (0.98–1.10)	0.17
CMV IgG concentration^d^ (per 50 U/mL)	0.94 (0.91–0.97)	0.0003	0.97 (0.94–1.01)	0.14	0.98 (0.95–1.02)	0.38
PI use at time of CT	0.94 (0.67–1.32)	0.73	1.18 (0.79–1.75)	0.42	1.17 (0.77–1.76)	0.45
NRTI use at time of CT	2.05 (0.92–5.19)	0.098	—	—	—	—
NNRTI use at time of CT	0.79 (0.58–1.08)	0.14	0.73 (0.51–1.06)	0.097	0.74 (0.51–1.08)	0.12
INSTI use at time of CT	1.11 (0.78–1.55)	0.56	1.09 (0.73–1.63)	0.66	1.10 (0.73–1.66)	0.63
TA/ddI exposure^c^, ^e^	0.46 (0.33–0.62)	< 0.0001	0.39 (0.27–0.56)	< 0.0001	0.54 (0.37–0.79)	0.0014
IDV/LPV/SQV exposure^c^, ^e^	0.47 (0.33–0.66)	< 0.0001	0.41 (0.28–0.60)	< 0.0001	0.53 (0.35–0.79)	0.0020

*Note:* No adjusted odds ratios were calculated for NRTI use, as all 53 PWH with thymus Grade 3 were using NRTIs.

Abbreviations: AIDS, acquired immunodeficiency syndrome; ART, antiretroviral therapy; CMV, cytomegalovirus; ddI, didanosine; IDV, indinavir; INSTI, integrase strand transfer inhibitor; LPV, lopinavir; NNRTI, non‐nucleoside reverse transcriptase inhibitor; NRTI, nucleoside reverse transcriptase inhibitor; PI, protease inhibitor; SQV, saquinavir; VL, viral load.

^a^ Adjusted for sex, pack‐years, BMI, waist‐to‐hip ratio, physical activity, and weekly alcohol intake.

^b^ Adjusted for sex, pack‐years, BMI, waist‐to‐hip ratio, physical activity, weekly alcohol intake, and age.

^c^ BMI violated the proportional odds assumption (Brant test *p* < 0.05) and was modeled as a non‐proportional term in the adjusted models.

^d^ Analysis restricted to the 617 PWH who were CMV IgG seropositive.

^e^ Models for TA/ddI and IDV/LPV/SQV exposure were not adjusted for waist‐to‐hip ratio, as it was considered a potential mediator.

**FIGURE 2:**
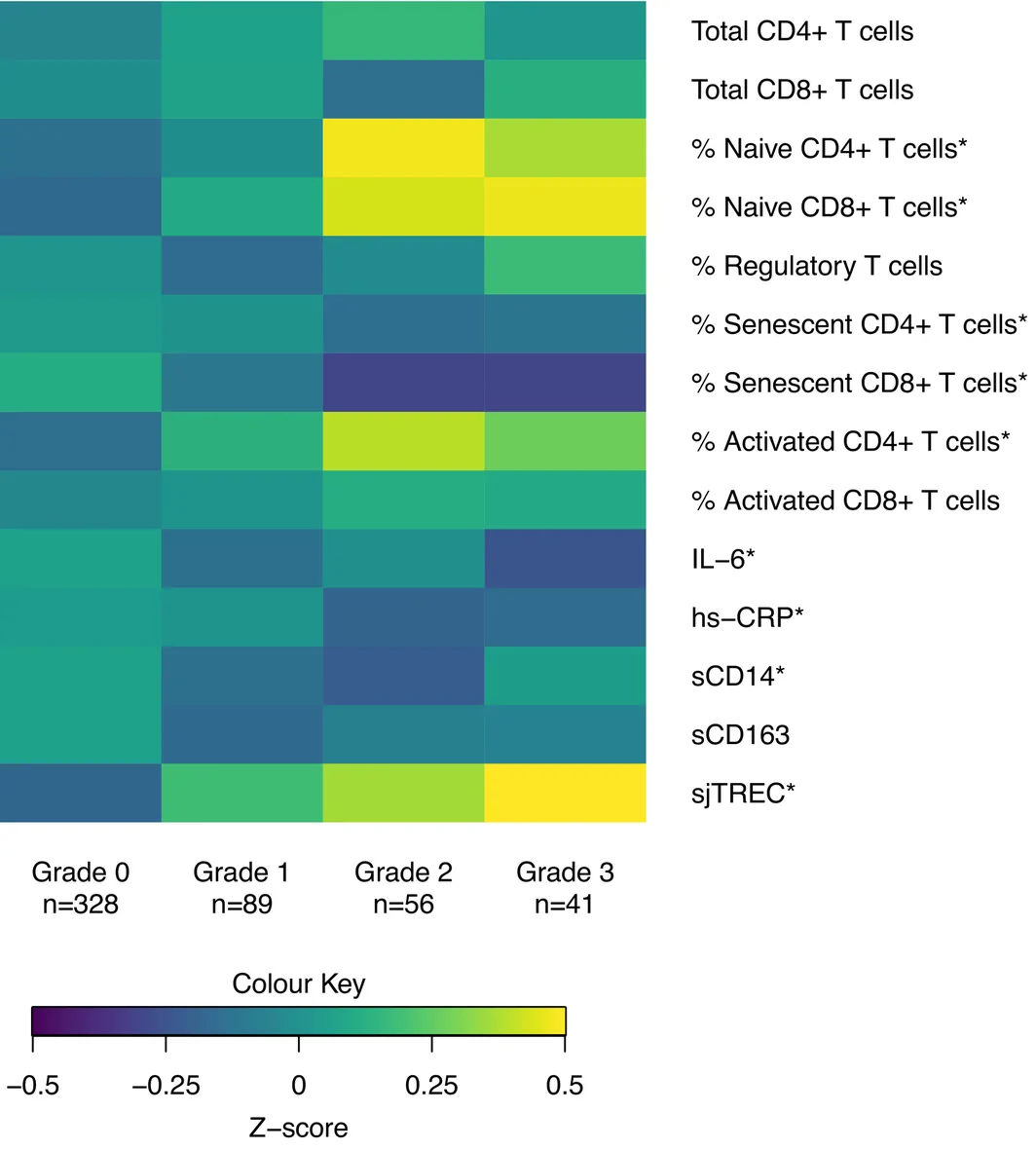
Heatmap of mean standardized biomarker levels by thymus grade, in PWH with complete markers. Heatmap depicting mean standardized biomarker levels by thymus grade, in PWH with complete biomarkers. The color displayed in each cell of the heatmap indicates the mean standardized level of the measured biomarker (i.e., z‐score) within the thymus grade group. Asterisks (*) denote biomarkers that differed significantly across thymus grades (*p* < 0.05). Abbreviations: Hs‐CRP, high sensitivity C‐reactive protein; IL‐6, interleukin 6; sCD14, soluble CD14; sCD163, soluble CD163, sjTREC, signal joint T‐cell receptor excision circle.

Among the 514 PWH (79%) with complete biomarker data, several markers differed significantly by thymus grade (Table S3). The proportions of naïve CD4^+^ and CD8^+^ T cells, as well as activated CD4^+^ T cells, as well as sjTREC content all increased as thymus grade increased (all *p* < 0.0001; Figure 2). In contrast, the proportions of senescent CD4^+^ (*p* = 0.0012) and CD8^+^ (*p* = 0.0005) T cells, as well as levels of the inflammatory markers IL‐6 (*p* < 0.0001) and hs‐CRP (*p* < 0.0001), all decreased as thymus grade increased. No significant differences across thymus grades were observed regarding total CD4^+^ (*p* = 0.27) and CD8^+^ T‐cell (*p* = 0.18) counts, regulatory T cells (*p* = 0.18), activated CD8^+^ T cells (*p* = 0.50), or levels of the innate immune activation markers sCD14 (*p* = 0.046) and sCD163 (*p* = 0.17).

## 4 Discussion

In this cross‐sectional study, we found that living with HIV was independently associated with having more thymic soft tissue on chest CT compared to population controls. The strong positive correlation between thymus grade and thymic attenuation indicates that higher grades indeed reflect more thymic soft tissue. Furthermore, higher thymus grade was associated with higher sjTREC content, signaling that thymic soft tissue observed on CT consists, at least in part, of functional lymphoid tissue capable of T‐cell receptor rearrangement and production of mature naïve T cells. These findings challenge the assumption that people with HIV experience a more pronounced loss in thymic function than those without HIV and align with our hypothesis that HIV infection may result in compensatory attenuation of thymic involution compared to its normal age‐related trajectory.

In line with prior studies in both the general population and in PWH, the majority of participants showed complete fatty replacement of thymic soft tissue (Guaraldi et al. 2019; Sandstedt et al. 2023). However, we show that fewer PWH had complete fatty replacement of the thymus compared to population controls. The possibility that thymic involution may be attenuated in some PWH was first raised by a small CT study from the mid‐1990s, before the widespread availability of combination ART (McCune et al. 1998). Our findings extend this earlier work by demonstrating, in a substantially larger and more contemporary cohort of PWH and matched population controls, that the association between HIV and preserved thymic soft tissue persists long after the initiation of effective antiretroviral treatment, even after adjustment for demographic and lifestyle factors. Furthermore, we show that PWH with higher thymus grades had significantly higher proportions of naïve T‐cells, lower proportions of senescent T‐cells, and lower circulating levels of IL‐6 and hs‐CRP. Together, these findings suggest that preserved thymic soft tissue in PWH is functionally relevant and may contribute to reduced immunosenescence and inflammaging. However, given the cross‐sectional design, we cannot exclude the possibility of reverse causality. Inflammation has been implicated as a driver of age‐related thymic involution through proinflammatory cytokine‐induced thymic epithelial cell senescence and loss of thymocytes, suggesting a bidirectional relationship between thymic involution and inflammaging (Liang et al. 2022; Santamaria and Irla 2026).

While we observed a positive association between sjTREC content and thymus grade, a considerable proportion of the samples fell below the lower limit of detection of the assay, limiting the interpretability of this finding. This is likely attributable, at least in part, to the relatively older age of participants, as T‐cell output is reduced in the aged thymus due to several factors, such as alterations in bone marrow progenitors and age‐associated changes in the thymic stromal environment (Palmer 2013). In addition, sjTRECs were measured in buffy coat samples, which contain most of the circulating leukocytes. Of these cells, only a minority consist of T‐cells, which will have diluted the sjTREC signal. Despite this limitation, both the proportion of participants with detectable sjTRECs and the median sjTREC value increased incrementally with thymus grade, and higher thymus grades were characterized by higher proportions of naïve T‐cells, suggesting that the sjTREC levels do correlate with active output of mature T‐cells from the thymus.

Evidence from prior studies suggests that thymic function may have implications for long‐term health. In the general population, all‐cause mortality and the risk of cancer are higher among patients who have undergone thymectomy than among controls (Kooshesh et al. 2023). A very recent study further demonstrated that better thymic health was consistently associated with lower all‐cause mortality, reduced lung cancer incidence, and lower cardiovascular mortality (Bernatz et al. 2026). In PWH, higher thymus grade has been cross‐sectionally associated with a lower prevalence of metabolic syndrome and frailty (Guaraldi et al. 2019). We also found in a previous study that PWH with higher thymic output and lower inflammation had fewer comorbidities over time (Vanbellinghen et al. 2024). While PWH are known to have a higher prevalence of aging‐related comorbidities than the general population, these findings suggest that the subgroup of PWH with preserved thymic output may be relatively protected, possibly through reduced chronic inflammation, a known driver of comorbidity risk in this population.

Although several HIV‐related variables were associated with thymus grade (including CD4 nadir, age at diagnosis, and time since diagnosis), these did not explain the observed association between HIV and higher thymus grades. This suggests that the higher thymus grades observed in PWH may reflect a response to peripheral T‐cell depletion or to HIV‐induced thymic damage, beyond disease severity or treatment regimen. One possible explanation could be endogenous thymic regeneration following suppression of HIV replication after ART initiation. The thymus has the capacity to regenerate after damage caused by factors such as infection or glucocorticoids or even following thymectomy in infants (Granadier et al. 2025; van Gent et al. 2011). Such thymic regrowth has indeed been documented in adult PWH 24 weeks after initiation of ART (Rubio et al. 2002). Younger age is the most important predictor of thymic regeneration, likely reflecting the presence of residual thymic tissue with functional regenerative capacity (Sun et al. 2016). In our analysis, younger age at HIV diagnosis was indeed strongly associated with higher thymus grade.

The observed association between TA/ddI exposure and lower thymus grade may reflect the known adverse effects of these drugs on body fat distribution (Gelpi, Afzal, et al. 2019; Gelpi, Knudsen, et al. 2019). Notably, exposure to first generation protease inhibitors (IDV/LPV/SQV), which are associated with lipohypertrophy (Carr et al. 1998), was similarly associated with lower thymus grade. When TA/ddI and first‐generation protease inhibitors were included simultaneously in the model, their effect sizes were attenuated but remained significant, suggesting that the association of each with lower thymus grade is not solely explained by co‐exposure to the other. Another explanation for the observed association is that these older antiretrovirals may serve as a proxy for an earlier treatment era with longer periods of untreated HIV infection, leading to destruction of thymocytes and consequently thymic atrophy (Luo et al. 2021).

Major strengths of this study include the largest sample size to date for such a comparative study on thymic involution, the use of an age‐ and sex‐matched control group, and the application of a standardized thymic grading method with strong intra‐ and interobserver agreement between raters. In addition, we measured peripheral markers of thymic output in nearly all participants and assessed an extensive set of immune and inflammatory markers in PWH. Limitations include the cross‐sectional design, which prevents assessment of changes in thymus grade over time and precludes inference about causality. The cohort consisted mainly of men of European descent, limiting generalisability to women and to the broader global population of people living with HIV. It should also be taken into account that T‐cell phenotyping was performed on cryopreserved PBMCs. While cryopreservation enables batch analysis and thereby reduces inter‐assay variability, freezing and thawing may introduce cell stress that can affect factors such as post‐thaw viability, differential recovery of T‐cell subsets, and the expression of cell‐surface markers. In this study, potential effects were minimized through the use of cryoprotective freezing medium, controlled‐rate freezing, and a standardized thawing protocol, and viability was within an acceptable range for immunophenotyping (median 75%, IQR 69%–87%). Although some influence of cryopreservation on T‐cell distribution cannot be ruled out, the distribution of T‐cells across thymic groups was comparable to that reported by Sandstedt et al. in the general population using fresh whole blood samples, suggesting that our findings are unlikely to be substantially affected by cryopreservation (Sandstedt et al. 2023). Finally, we cannot rule out survival bias, as PWH with residual thymic tissue at the time of their HIV infection may have been more likely to survive HIV‐related illness and thus be included in the cohort.

In conclusion, PWH on long‐term ART experience higher thymus grades than age‐ and sex‐matched controls from the general population, possibly reflecting thymic regeneration following ART initiation. Higher thymus grade in PWH was associated with a higher proportion of circulating naïve and lower proportion of senescent T‐cells, as well as less systemic inflammation. This suggests that thymic function may mitigate persistent immune activation and inflammation in such individuals with HIV and thereby could benefit their long‐term health.

## Author Contributions

Conceptualisation: M.C.V., A.B., S.D.N., and P.R. Data curation: M.C.V., A.O.J., and M.A.S.‐Z. Formal analysis: M.C.V. and A.B. Funding acquisition: B.G.N. and S.D.N. Investigation: M.C.V., A.O.J., and N.K. Methodology: M.C.V., A.B., A.D.K., and T.S.K. Project administration: A.O.J., M.A.S.‐Z., P.E.S., M.H.C.P., A.F.L., and A.D.K. Resources: M.A.S.‐Z., J.T.K., K.F.K., P.E.S., M.H.C.P., A.F.L., B.G.N., S.A., N.K., and S.D.N. Supervision: J.T.K., S.D.N., and P.R. Validation: T.S.K. Visualization: M.C.V. Writing – original draft: M.C.V., A.B., P.R., and N.K. Writing – review and editing: all authors. All authors critically revised the paper and had final responsibility for the decision to submit for publication. M.C.V. and A.B. accessed and verified the underlying data reported in the manuscript.

## Funding

This work was supported by Novo Nordisk, Independent Research Fund Denmark, Copenhagen University Hospital Rigshospitalet Research Council, Danish Heart Association, Aase og Ejnar Danielsen Foundation, and the Copenhagen County Foundation.

## Conflicts of Interest

A.B. reports lecture honoraria from Gilead Sciences. M.A.S.‐Z. reports salary support from Rigshospitalet Research Council, lecture honoraria from Gilead Sciences, and membership of the Rigshospitalet Research Council grant board. A.D.K. reports grants to their institution from Gilead Sciences, Rigshospitalet Research Council, and Region Hovedstaden Research Council. K.F.K. reports research grants from AP Møller og Hustru Chastine McKinney Møllers Fond, Rigshospitalet Research Council, the University of Copenhagen, the Danish Heart Foundation, the Danish Agency for Science, Technology and Innovation by the Danish Council for Strategic Research, Novo Nordisk Foundation, Canon Medical Systems, and GE Healthcare. S.D.N. reports research grants from Rigshospitalet Research Council, Gilead Sciences, and Svend Andersen Foundation; salary support from the Independent Research Fund Denmark and Dr. Sofus Carl Emil Friis and Wife Olga Doris Friis Scholarship; a grant from Novo Nordisk Foundation unrelated to the present study; lecture honoraria from MSD and Gilead Sciences; participation on advisory boards for Gilead, MSD, and GSK; and membership of the Rigshospitalet Research Council grant board. P.R. reports investigator‐initiated research grants to their institution from Gilead Sciences, Merck & Co, and ViiV Healthcare; partial financial support from Gilead Sciences to attend an international conference; and remuneration to self for participation on the NIH/NIAID HIV vaccine trials Data Safety Monitoring Board. All other authors report no conflicts of interest.

## Supporting information


**Table S1:** Missingness in variables used in regression analyses, by HIV status.
**Table S2:** Thymic attenuation and size measurements by thymus grade, for participants with thymus Grade ≥ 1.
**Table S3:** Biomarkers in PWH with complete biomarker data, by thymus grade.
**Figure S1:** Flow chart of participant selection.
**Figure S2:** Standard curve used for sjTREC quantification.
**Figure S3:** Representative CT images in the axial plane of thymus Grades 0 to 3.
**Figure S4:** Thymic attenuation (HU) across thymus Grades 1 to 3 in PWH and population controls.

## Data Availability

The data used for this study are not publicly available as they contain information that could compromise participant privacy and consent but are available from the corresponding author on reasonable request to the extent allowed by data protection legislation. Access to non‐anonymised data will require approval from the data protection office.
