# Supplementary material for: Thymic Involution and Function in People With HIV and General Population Controls: A Cross‐Sectional Analysis From Two Danish Cohort Studies
Source: Aging Cell. 2026 Sep 23;25(10):e70723. doi: 10.1111/acel.70723 (PMC13598926; doi:10.1111/acel.70723)
Supplement: Supplementary file 1 [file ACEL-25-e70723-s001.docx]

**Supporting Data**

**Table of contents**

| Supplementary Table 1. Missingness in variables used in regression analyses, by HIV status | P2 |
| --- | --- |
| Supplementary Figure 1. Flow chart of participant selection | P3 |
| Supplementary Figure 2. Standard curve used for sjTREC quantification | P4 |
| Supplementary Figure 3. Representative CT images in the axial plane of thymus grades 0 to 3 | P5 |
| Supplementary Table 2. Thymic attenuation and size measurements by thymus grade, for participants with thymus grade ≥ 1 | P6 |
| Supplementary Figure 4. Thymic attenuation (HU) across thymus grades 1 to 3 in PWH and population controls | P7 |
| Supplementary Table 3. Biomarkers in PWH with complete biomarker data, by thymus grade | P8 |

**Supplementary Table 1. Missingness in variables used in regression analyses, by HIV status**

|  | PWH (n = 654) | Population Controls (n= 637) |
| --- | --- | --- |
| Age | 0 (0%) | 0 (0%) |
| Sex | 0 (0%) | 0 (0%) |
| European descent | 9 (1%) | 11 (2%) |
| BMI | 3 (0%) | 7 (1%) |
| Waist-to-hip ratio | 19 (3%) | 11 (2%) |
| Pack years | 25 (4%) | 11 (2%) |
| Weekly alcohol consumption | 54 (8%) | 13 (2%) |
| Physical activity | 29 (4%) | 9 (1%) |
| Age at HIV diagnosis | 9 (1%) | … |
| Time since HIV diagnosis | 9 (1%) | … |
| HIV RNA <200 copies/mL | 5 (1%) | … |
| HIV RNA at start ART | 131 (20%) | … |
| Prior AIDS | 1 (0%) | … |
| CD4 nadir | 12 (2%) | … |
| CMV IgG concentration | 1 (0%) |  |
| PI use | 9 (1%) | … |
| NRTI use | 9 (1%) | … |
| NNRTI | 9 (1%) | … |
| INSTI use | 9 (1%) | … |
| TA/ddI exposure | 0 (0%) | … |
| IDV/LPV/SQV exposure | 0 (0%) | … |

*Note.* Data is reported as n (%)

Abbreviations: AIDS, acquired immunodeficiency syndrome; ART, antiretroviral therapy; BMI, body mass index; CMV, cytomegalovirus; ddI, didanosine; IDV, indinavir; INSTI, integrase strand transfer inhibitor; LPV, lopinavir; NRTI, nucleoside reverse transcriptase inhibitor; NNRTI, non-nucleoside reverse transcriptase inhibitor; PI, protease inhibitor; RNA, ribonucleic acid; SQV, saquinavir; TA, thymidine analogue.

**Supplementary Figure 1.** **Flow chart of participant selection**

*Note*: Flow chart of participant selection. Abbreviations: CGPS, Copenhagen General Population Study; COCOMO, Copenhagen Comorbidity in HIV Infection study; CT, computed tomography; PWH, people with HIV

**Supplementary Figure 2.** **Standard curve used for sjTREC quantification**

*Note:* The x-axis shows the log_10_-transformed sjTREC copy number, and the y-axis shows the Ct value (mean ± SD). Abbreviations: Ct, cycle threshold.

**Supplementary Figure 3.** **Representative CT images in the axial plane of thymus grades 0 to 3**

A. Grade 0 in 42-year-old male PWH	B. Grade 0 in 67-year-old male population control

C. Grade 1 in 51-year-old male PWH	D. Grade 1 in 42-year-old male population control

E. Grade 2 in 52-year-old male PWH	F. Grade 2 in 45-year-old male population control

G. Grade 3 in 43-year-old male PWH	H. Grade 3 in 43-year-old male population control

**Supplementary Table 2**. **Thymic attenuation and size measurements by thymus grade, for participants with thymus grade ≥ 1**

|  | Thymus grade 1  (n=218) | Thymus grade 2  (n=118) | Thymus grade 3  (n=80) | Τau-b | p*-value* |
| --- | --- | --- | --- | --- | --- |
| Attenuation (HU),  mean (95% CI) | -54.7 (-56.7, - 52.7) | -31.7 (-34.5, -28.9) | -0.30 (-4.16, 3.55) | 0.646 | <0.0001 |
| AP diameter (mm),  median (IQR) | 14.4 (12.3-17.9) | 13.9 (11.3-16.7) | 14.2 (11.2-16.5) | -0.085 | 0.026 |
| Transverse diameter (mm), median (IQR) | 24.3 (20.1-30.3) | 22.6 (17.3-26.8) | 21.2 (16.7-26.3) | -0.138 | 0.0003 |

*Note.* Abbreviations: AP, anteroposterior; CI, confidence interval; HU, Hounsfield unit

**Supplementary Figure 4.**

*Thymic attenuation (HU) across thymus grades 1 to 3 in PWH and population controls*

*Note.* Individual points represent individual participants, with black circles indicating people with HIV and grey circles indicating population controls. Trendlines represent linear regression fits, with shaded bands representing 95% confidence intervals.

Abbreviations: HU, Hounsfield units; PWH, people with HIV; τb, Kendall’s tau-b correlation coefficient.

**Supplementary Table 3**. **Biomarkers in PWH with complete biomarker data, by thymus grade**

|  | Thymus grade 0  n=328 (64%) | Thymus grade 1  n= 89 (17%) | Thymus grade 2  n= 56 (11%) | Thymus grade 3  n= 41 (8%) | p-value |
| --- | --- | --- | --- | --- | --- |
| Total CD4^+^ T cells, cells/ µl | 660 (510-850) | 690 (545-910) | 738 (576-952) | 660 (549-845) | 0.27 |
| Total CD8^+^ T cells, cells/ µl | 816 (620-1100) | 878 (704-1200) | 775 (562-972) | 840 (678-1300) | 0.18 |
| % Naïve CD4^+^ T cells | 37 (27-47) | 39 (31-47) | 48 (36-58) | 47 (38-56) | <0.0001 |
| % Naïve CD8^+^ T cells | 21 (14-30) | 28 (17-35) | 31 (23-40) | 35 (24-42) | <0.0001 |
| % Regulatory T cells | 4.3 (3.3-5.7) | 3.9 (3.2-5.2) | 4.4 (3.4-6.0) | 4.8 (4.0-5.7) | 0.18 |
| % Senescent CD4^+^ T cells | 0.34 (0.13-0.74) | 0.32 (0.13-0.72) | 0.18 (0.07-0.39) | 0.17 (0.10-0.36) | 0.0012 |
| % Senescent CD8^+^ T cells | 18 (12-25) | 16 (10-21) | 13 (9-18) | 13 (8-21) | 0.0005 |
| % Activated CD4^+^ T cells | 7.6 (5.2-10.1) | 7.9 (6.6-11.0) | 10.0 (7.4-12.0) | 8.2 (7.0-11.6) | <0.0001 |
| % Activated CD8^+^ T cells | 23 (17-30) | 25 (19-30) | 26 (19-31) | 24 (19-32) | 0.50 |
| IL-6, pg/ml | 1.7 (1.2-2.7) | 1.2 (0.9-1.7) | 1.2 (0.9-1.9) | 1.2 (0.8-1.6) | <0.0001 |
| hs-CRP, mg/l | 1.4 (0.8-2.9) | 0.9 (0.5-2.0) | 0.9 (0.5-1.8) | 0.7 (0.4-1.8) | <0.0001 |
| sCD14, ng/ml | 3647 (3165-4363) | 3467 (2905-4185) | 3343 (3010-4066) | 3776 (3047-4365) | 0.046 |
| sCD163, ng/ml | 913 (670-1252) | 801 (626-1143) | 768 (644-1033) | 860 (679-1090) | 0.17 |
| sjTREC^1^, log copies/ng DNA | 5.0 (5.0-5.0) | 5 (5.0-6.1) | 5 (5.0-6.3) | 5.6 (5.0-6.3) | <0.0001 |

Data are presented as n (%) or median (IQR).

Abbreviations: CD, cluster of differentiation; hs-CRP, high-sensitivity C-reactive protein; IL, interleukin; sCD, soluble cluster of differentiation; sjTREC, signal joint T-cell receptor excision circle

^1^Samples below the LLD were assigned a value of 5 log sjTREC count/ng DNA for the calculation of medians and interquartile ranges. The proportion of participants with sjTREC levels above the lower limit of detection (LLD) was 18% (58/328), 34% (30/89), 41% (23/56), and 51% (21/41) for thymus grades 0, 1, 2, and 3, respectively.
